# The Effects of Sleep Hypoxia on Coagulant Factors and Hepatic Inflammation in Emphysematous Rats

**DOI:** 10.1371/journal.pone.0013201

**Published:** 2010-10-06

**Authors:** Jing Feng, Qing-shan Wang, Ambrose Chiang, Bao-yuan Chen

**Affiliations:** 1 Respiratory Department, Tianjin Medical University General Hospital, Tianjin, China; 2 Division of Pulmonary and Critical Care Medicine, Duke University Medical Center, Durham, North Carolina, United States of America; 3 Institute of Toxicology, Shandong University, Jinan, Shandong, China; University of California, Merced, Please select

## Abstract

**Objectives:**

To develop a sleep hypoxia (SH) in emphysema (SHE) rat model and to explore whether SHE results in more severe hepatic inflammation than emphysema alone and whether the inflammation changes levels of coagulant/anticoagulant factors synthesized in the liver.

**Methods:**

Seventy-five rats were put into 5 groups: SH control (**SHCtrl**), treated with sham smoke exposure (16 weeks) and SH exposure (12.5% O_2_, 3 h/d, latter 8 weeks); emphysema control (**ECtrl**), smoke exposure and sham SH exposure (21% O_2_); short SHE (**SHEShort**), smoke exposure and short SH exposure (1.5 h/d); mild SHE (**SHEMild**), smoke exposure and mild SH exposure (15% O_2_); standard SHE (**SHEStand**), smoke exposure and SH exposure. Therefore, ECtrl, SHEShort, SHEMild and SHEStand group were among emphysematous groups. Arterial blood gas (ABG) data was obtained during preliminary tests. After exposure, hepatic inflammation (interleukin -6 [IL-6] mRNA and protein, tumor necrosis factor α [TNFα] mRNA and protein) and liver coagulant/anticoagulant factors (antithrombin [AT], fibrinogen [FIB] and Factor VIII [F VIII]) were evaluated. SPSS 11.5 software was used for statistical analysis.

**Results:**

Characteristics of emphysema were obvious in emphysematous groups and ABGs reached SH criteria on hypoxia exposure. Hepatic inflammation parameters and coagulant factors are the lowest in SHCtrl and the highest in SHEStand while AT is the highest in SHCtrl and the lowest in SHEStand. Inflammatory cytokines of liver correlate well with coagulant factors positively and with AT negatively.

**Conclusions:**

When SH is combined with emphysema, hepatic inflammation and coagulability enhance each other synergistically and produce a more significant liver-derivative inflammatory and prothrombotic status.

## Introduction

Chronic obstructive pulmonary disease (COPD), in which chronic systemic inflammation is a central component, is a growing global epidemic that is particularly important in developing countries [1]. It is expected that in 2020 COPD will become the third leading cause of death worldwide [2]. In humans, COPD includes two major subtypes, chronic bronchitis and emphysema. The emphysema subtype seems to present more obvious systemic inflammation, suggesting that it may be more prone to systemic consequences of COPD compared to subtype without emphysema [3]. Sleep hypoxemia (SH) in The International Classification of Sleep Disorders: Diagnostic and Coding Manual 2 (ICSD-2) is defined as “an SpO_2_ (oxyhemoglobin saturation) during sleep of >30% of total sleep time with an SpO_2_ of <90% in subject with a baseline awake SpO_2_ of ≥90% ”[4], [5]. The phenomenon of SH complicating emphysema has been recognized for at least 50 years [6] and has been noted in polysomnography (PSG) studies of emphysematous patients recently [7]. Because of a number of factors, mainly including: alveolar hypoventilation, airway obstruction, hyperinflation, respiratory muscle dysfunction, blunted ventilatory responses to hypoxia and ventilation-perfusion mismatch, emphysematous patients run a high risk of developing SH [8]–[10]. Compared to their non-SH brethren, emphysematous patients with SH have greater degrees of pulmonary hypertension and cor pulmonale [11], require more frequent hospitalizations, sustain higher mortality rates [12]–[15] and demonstrate systemic evidence of pronounced inflammatory activation [16] which may cause inflammatory damages to the liver as well.

Increased risk of thrombotic events occurs in COPD [17]. Venous thromboembolism (VTE) occurs with increased frequency in patients with COPD which may be due to hypercoagulability as a result of hypoxia [18]. Most of the coagulant factors and anticoagulant factors are synthesized in liver and among them, antithrombin (AT), fibrinogen (FIB) and Factor VIII (F VIII) play very important roles in coagulation cascade. AT, as a central anticoagulant in mammalian circulation system, binds and inactivates the serine proteases (Factors XIa, IXa, Xa, and thrombin) directly [19]; FIB, a soluble glycoprotein, is converted by thrombin into fibrin during blood coagulation and is then cross linked by factor XIII to form blood clot [20]; F VIII, an essential blood clotting factor also known as anti-hemophilic factor, is bound to von Willebrand factor (vWF) to form a stable complex in circulating blood and once activated by thrombin it dissociates from the complex to interact with Factor IXa and participates in the coagulation cascade for crosslinking into a blood clot. Without the protection of vWF, activated F VIII is proteolytically inactivated and cleared from the blood stream quickly [21].

Hypoxia induces systemic inflammation and acts as an aggravating factor of liver inflammation [22]–[24]. Compared with isolated emphysema, it has not yet been clearly elucidated whether SH in emphysema can cause more obvious inflammation in liver and whether the inflammation changes levels of coagulant/anticoagulant factors synthesized in the liver. To examine this, we exposed emphysematous rats (the pre-existing emphysema was formed through smoke exposure for 16 weeks) to hypoxia during sleep in a custom-made chamber therefore developing a novo rat model of SH in emphysema (SHE); with this model the inflammatory cytokines of the liver was studied and the four important coagulant/anticoagulant factors just mentioned above were measured. The inflammatory cytokines we measured in this study included interleukin -6 (IL-6, a classic pro-inflammatory cytokine) mRNA and protein levels, tumor necrosis factor α (TNFα, a classic pro-inflammatory cytokine) mRNA and protein levels.

## Methods

The overall experimental protocol is hypoxia exposure during sleep on the base of pre-existing emphysema which is caused by 16 weeks of smoke exposure followed by exploring inflammatory and coagulant/anticoagulant factors of the liver. Institutional Review Board of Tianjin Medical University General Hospital approved the ethical and methodological aspects of the investigation (TMU IRB Approving Number: EA-20080002.).

### Animal cigarette smoke exposure

Seventy-five male Wistar rats weighing 150∼180 g at age of 7 weeks (provided by Model Animal Center of Radiological Medicine Research Institute, Chinese Academy of Medical Science, license No.: SCXK Tianjin 2005-0001) were divided into 5 groups of 15 according to exposure conditions. Animals were housed in standard laboratory cages (5 per cage) and allowed food and water ad libitum. Rats were passively exposed to the smoke of 15 commercial unfiltered cigarettes (Daqianmen™, Yunnan, China) for 30 minutes twice daily, in the morning (before 9 am) and in the evening (after 5 pm), for 16 weeks (W 1 to the end of W 16) continuously in a custom-made plexiglas chamber (0.6 m^3^ with 5 poles in side wall for ventilation, 15 rats per batch). Content of tar was 18 mg for every cigarette and concentration of smog was about 15% (V/V) within the housing chamber through burning of 5 cigarettes concurrently. Control rats were exposed to a sham environment otherwise the same condition except of cigarette burning [25].

### Animal electroencephalogram monitoring and SH exposure

A week prior to the experimental exposure, two stainless steel screws attached to insulated wires were implanted in the skull (from bregma: anteroposterior, +2 mm, mediolateral, −2 mm; and anteroposterior, −3 mm, mediolateral, +2 mm) of all recruited rats to record electroencephalogram (EEG) [26]. For electrophysiological recording, a lightweight shielded cable was connected to the plug on the rats' heads which permitted free movement of the rats within the cage. The signals were routed to an electroencephalograph (Nicolet Bravo, USA) so that sleep stages could be monitored conveniently and continuously. We designed a hypoxia pattern definitely different from intermittent hypoxia seen in previous literatures [27], [28] used in study on obstructive sleep apnea (OSA) and our focused hypoxia pattern was an “intermittent continuous hypoxia” often seen in SH of emphysematous patients [4], [5]. Briefly, a gas control delivery system [29] was designed to regulate the flow (2.5 L/min) of premixed gas (21% O_2_, 15% O_2_ or 12.5% O_2_) or clean air continuously into a customized SH housing chamber (volume about 10 L) to maintain a designated continuous hypoxia or normoxia environment. A series of programmable solenoids and flow regulators altered the fractional concentration of inspired oxygen through software edited with Visual C^++^ computer language that controlled the durations of hypoxia/normoxia cycles, which could divide total hypoxia time (1.5 h or 3 h) into 4 periods evenly (22.5 min or 45 min each) and distribute these hypoxia periods into physiological sleep time of Wistar rats identified by EEG (9 AM to 5 PM as estimated). The chamber was equipped with a humidifier, thermostat, and molecular sieve to maintain an inner temperature of 22 °C, humidity of ∼45% and a germfree circumstance. O_2_ and CO_2_ concentration monitors (Hamilton, Switzerland) were used to determine the environmental situation in the housing chamber for rats.

### Exposure protocol

Seventy-five male Wistar rats were seperated into 5 groups of 15 as follows: A. SH control group (**SHCtrl**), sham smoke exposure (W 1 to W 16) and true SH exposure (12.5% O_2_, 3 h, W 9 to W 16); B. Emphysema control group (**ECtrl**), true smoke exposure (W 1 to W 16) and sham SH exposure (21% O_2_, 3 h, W 9 to W 16); C. Short SHE group (**SHEShort**), smoke exposure (W 1 to W 16) and short SH exposure (12.5% O_2_, 1.5 h, W 9 to W 16); D. Mild SHE group (**SHEMild**), smoke exposure (W 1 to W 16) and mild SH exposure (15% O_2_, 3 h, W 9 to W 16); E. Standard SHE group (**SHEStand**), smoke exposure (W 1 to W 16) and SH exposure (12.5% O_2_, 3 h, W 9 to W 16). Therefore, the ECtrl, SHEShort, SHEMild and SHEStand group were expected emphysematous groups according to experimental design.

### Preliminary experiment to get the arterial blood gas (ABG)

Two days before the end of exposure period (end of W 16 – 2 d), 5 rats were selected randomly from each group to obtain ABG data. After anesthetization (10% chloral hydrate, 0.3 mL/100 g body weight intraperitoneally), the right femoral artery of selected rats was cannulated to obtain blood samples for monitoring ABG at anytime as necessary. At the end of treatment (end of W 16), arterial blood samples (0.7 mL each) were drawn under the following conditions:

Point 1: Wakefulness in **SHCtrl** group with clean air exposure (without emphysema, awake, 21% O_2_).

Point 2: Slow wave sleep period in **SHCtrl** group with clean air exposure (without emphysema, sleep, 21% O_2_).

Point 3: Slow wave sleep period in **SHCtrl** group with SH exposure (without emphysema, sleep, 12.5% O_2_).

Point 4: Wakefulness in **ECtrl** group with clean air exposure (emphysema, awake, 21% O_2_).

Point 5: Slow wave sleep period in **ECtrl** group with clean air exposure (emphysema, sleep, 21% O_2_).

Point 6: Slow wave sleep period in **SHEMild** group with SH exposure (emphysema, sleep, 15% O_2_).

Point 7: Slow wave sleep period in **SHEStand** group with SH exposure (emphysema, sleep, 12.5% O_2_).

For each blood sample collected, ABG analysis included measurements of partial oxygen pressure (PaO_2_), partial carbon dioxide pressure (PaCO_2_), arterial oxygen saturation (O_2_Sat) and pH value.

### Measurements for AT, FIB, F VIII and vWF

The remaining 10 rats from each group were used for following experiments at the end of exposure. Blood samples were obtained from tail veins using sodium citrate in order to perform AT, FIB, F VIII and vWF tests through an ACL 9000 automated coagulation analyzer (Beckman Coulter, CA) with proper reagent kits (Dade Behring, IL) in the core hospital laboratory. Activities of AT and F VIII were measured in percent to standard plasma activity and concentration; the concentration of FIB was expressed as mg/dL; and the concentration of vWF Ag was reported as percentage in relation to a photometric extinction of standard plasma.

### Histologic identification of emphysema

After anesthetization and isolation of lungs, all lung samples were inflated intrabronchially with 10% formalin at a constant transpulmonary pressure of 25 cm of formalin for at least 18 hours with samples impregnated wholly in formalin at the same time. Then lung blocks were fixed, embedded, cut and stained. Images were captured at 40 X and were saved as JPEG files [30]. Pictures were then given to pathologists with expertise in the pathology of emphysema for assessment.

### Measurements for IL-6 mRNA and protein levels, TNFα mRNA and protein levels

For liver tissue preparation, blocks from each rat were frozen in liquid nitrogen and stored at −80°C in order to analyze IL-6 and TNFα. The levels of IL-6 and TNFα gene expression were quantified using real time RT-PCR analysis. Total RNA was isolated from rat liver samples using Trizol reagent, followed by purification with RNeasy Mini Kit (Qiagen, MD). Total RNA (1 µg) was reverse transcribed with murine leukemia virus reverse transcriptase and random hexamer primers. Primers for IL-6 and TNFα were designed using Primer Express software (version 2.0, Applied Biosystems, CA). The primer sequences used in this study were as follows: IL-6, 5′-CTG ATT GTA TGA ACA GCG ATG-3′ (forward) and 5′-GAA CTC CAG AAG ACC AGA GC-3′ (reverse); TNFa, 5′-GAC CCT CAC ACT CAG ATC ATC TTC T-3′ (forward) and 5′-CCT CCA CTT GGT GGT TTG CT-3′ (reverse); GAPDH, 5′-CCT GGA GAA ACC TGC CAA GTA T-3′ (forward) and 5′-AGC CCA GGA TGC CCT TTA GT-3′ (reverse). The SYBR green DNA PCR kit (Applied Biosystems, CA) was used for real time PCR analysis. Gene expression data was expressed using cycle time values normalized with internal GAPDH, and relative differences between control and treatment groups were calculated and expressed as relative increases setting control as 100%. The frozen liver blocks were also homogenized in 100 mg tissue/ml cold lysis buffer (20 mM Tris, 0.25 M sucrose, 2 mM EDTA, 10 mM EGTA, 1% Triton X-100 and 1 tablet of Complete Mini protease inhibitor cocktail/10 ml, Roche Diagnostics, IN). Homogenates were centrifuged at 100,000× g for 30 min, supernatant was collected, and total protein levels were determined using BCA protein assay reagent kit (PIERCE, WI). The protein levels of liver IL-6 and TNFα were measured with IL-6 and TNFα commercial enzyme-linked immunosorbent assay (ELISA) kits (R&D Systems, MN).

### Statistical Analysis

SPSS 11.5 (SPSS Inc., Chicago, IL) software package was used for statistical analysis and illustration. Preliminary ABG data was obtained and is displayed as a descriptive analysis. One way analysis of variance (*ANOVA*) was performed for whole difference and *Bonferroni* post hoc multiple comparisons were used to evaluate differences between internal groups. Linear correlation analysis and linear fitting were performed for influential power estimating to see whether significant correlations exist between relevant parameters. Unless otherwise stated, values were reported as mean ± standard deviation (SD), and *P*<0.05 is considered statistically significant.

## Results

**Figure 1:**
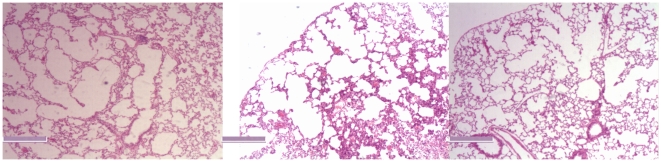
Pathologic pictures were obtained from emphysematous rats, stained with hematoxylin/eosin (H/E), and captured with 40 X light microscopy. Scale at left lower represents the length of 1000 µm.

Figure 1 shows the pathologic pictures selected randomly from emphysematous rats. Pathologic characteristics of emphysema, though may be in different severities among groups, were obvious in the emphysematous rats, including inflammatory cell infiltration, increasing of mean linear intercept and decreasing of mean alveolar number per microscope field. (detail not provided in this article) [31]–[33].

Table 1 summarizes the ABG results obtained from preliminary experiments two days before the end of exposure period. The values in this table are descriptive, not for comparing between groups. PaO_2_ value of SHCtrl in SH(12.5%) is 55.083±0.3971 mmHg and PaO_2_ values of ECtrl in wakefulness, sleep, SH(15%) and SH(12.5%) are 84.467±3.6092, 73.400±2.1615, 54.617±2.0371 and 46.567±2.2844 mmHg, respectively.

**Table 1:** Descriptive ABG results from preliminary tests.

	SHCtrl group			ECtrl group			
	Wakefulness	Sleep	SH (12.5%)	Wakefulness	Sleep	SH (15%)	SH (12.5%)
**PaO_2_ (mmHg)**	110.600±4.2000	94.283±2.3353	55.083±0.3971	84.467±3.6092	73.400±2.1615	54.617±2.0371	46.567±2.2844
**PaCO_2_ (mmHg)**	39.500±2.5322	41.017±2.9390	35.967±0.2944	44.150±2.1980	49.017±2.6095	47.500±2.0871	49.650±0.9894
**O_2_Sat (%)**	99.083±0.2483	97.000±0.2000	85.050±1.1362	94.783±1.5484	91.917±0.6524	85.850±1.0173	83.450±1.7593
**pH**	7.40683±0.015523	7.41333±0.017580	7.41983±0.001941	7.37200±0.019698	7.35817±0.005776	7.35283±0.007387	7.35383±0.004622

PaO_2_: arterial oxygen partial pressure, PaCO_2_: arterial carbon dioxide partial pressure, O_2_Sat: arterial oxyhemoglobin saturation.

Table 2 demonstrates that all measured inflammatory parameters and coagulant factors are the lowest and AT (an anticoagulant factor) the highest in SHCtrl. Inflammatory cytokines and coagulant factors in SHEStand are the highest except for AT and vWF. AT in SHEStand has a tendency to be the lowest though does not attain statistical significance. For **AT**: SHCtrl is the highest; ECtrl is lower than SHCtrl, higher than SHEStand and not different with others; SHEStand is lower than SHCtrl and Ectrl but not different with SHEShort and SHEMild. For **vWF**: SHCtrl is the lowest; ECtrl is higher than SHCtrl, not different with SHEMild and lower than others; SHEStand is not different with SHEShort and higher than others. For **FIB**, **F VIII**, **IL-6 mRNA**, **IL-6 protein**, **TNFα mRNA** and **TNFα protein**: SHCtrl is the lowest; ECtrl is higher than SHCtrl and lower than SHEStand; SHEStand is the highest. More detail comparing results are shown in Table 2.

**Table 2:** Coagulant/anticoagulant factors and hepatic inflammation status.

	SHCtrl	ECtrl	SHEShort	SHEMild	SHEStand
**Whole blood coagulation**					
**AT: A (%)**	116.2±2.53*	107.39±8.045†	103.72±6.31‡	101.8±4.278§	99.04±3.986||
**FIB (mg/dL)**	151.55±6.2789*	177.46±7.2485/	189.98±5.2845/	182.7±2.7761#	198.4±7.369*
**F VIII: C (%)**	200.36±4.8754*	227.78±10.3779∧	232.26±4.1727∧	242.53±14.5011∧	303.25±32.9323*
**vWF: Ag (%)**	56.02±1.4650*	64.45±1.72/	70.2±8.6665@	66.21±3.2102#	71.96±1.8506^x^
**Hepatic inflammtion cytokines**					
**IL-6 mRNA (standardized)**	99.990±5.0538*	142.140±6.1437*	226.030±7.3423*	244.870±6.5323*	288.260±5.7514*
**IL-6 protein (ng/100 mg protein)**	10.390±1.1060*	12.470±1.2508∧	13.110±1.5337∧	12.990±1.2405∧	19.040±1.6788*
**TNFα mRNA (standardized)**	100.000±6.1601*	181.430±4.4086*	227.790±8.7492*	252.700±9.8080*	295.090±8.5188*
**TNFα protein (ng/100 mg protein)**	9.940±0.5379*	12.230±0.4244*	13.430±0.5908/	14.010±0.7125^x^	16.860±0.3373*

**Note:**

Unless otherwise stated, there is significant difference in *Bonferroni* post hoc multiple comparisons compared with other groups.

* different with others;

† not different with SHEShort and SHEMild;

‡ not different with ECtrl, SHEMild and SHEStand;

§ not different with ECtrl, SHEShort and SHEStand;

|| not different with SHEShort and SHEMild;

/ not different with SHEMild;

# not different with ECtrl and SHEShort;

∧ no difference among ECtrl, SHEShort and SHEMild;

@ not different with SHEMild and SHEStand;

x not different with SHEShort.

**Figure 2:**
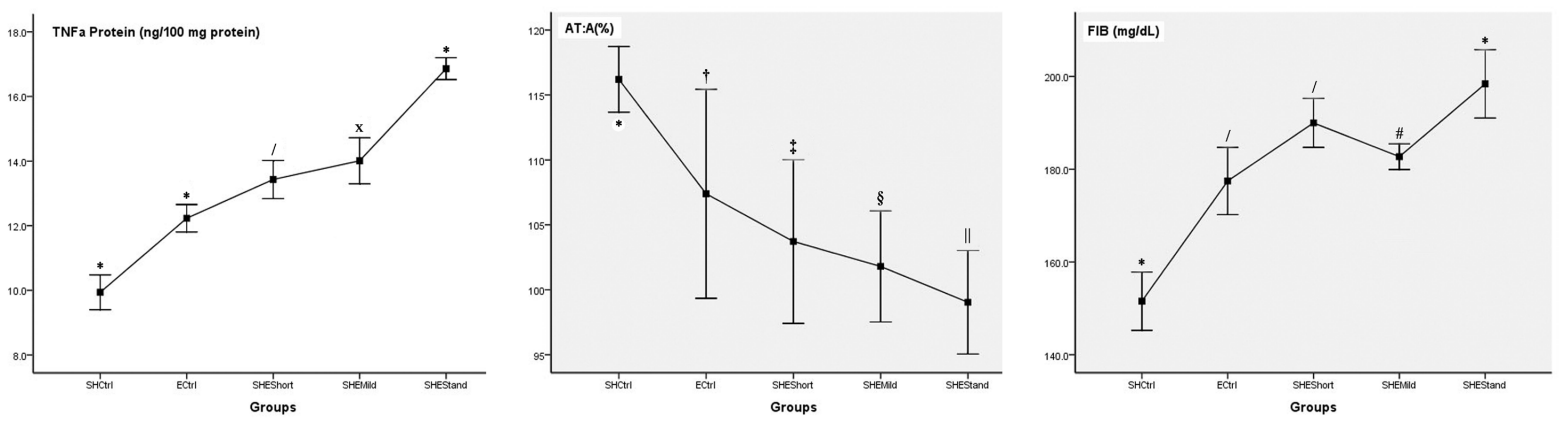
TNFα protein and FIB increase from SHCtrl to SHEStand while AT shows the obverse tendency. Error bars: +/− 1 SD. *P*<0.05 is considered statistically significant in *Bonferroni* post hoc multiple comparisons between internal groups. Unless otherwise stated, there is significant difference compared with other groups. * different with others; /not different with SHEMild; x not different with SHEShort; † not different with SHEShort and SHEMild; ‡ not different with ECtrl, SHEMild and SHEStand; § not different with ECtrl, SHEShort and SHEStand; || not different with SHEShort and SHEMild; # not different with ECtrl and SHEShort.

For demonstrating clearly, Figure 2 plots this data showing that inflammatory cytokines of liver (represented with TNFα) and coagulant factors (represented with FIB) increase obviously from left to right (from SHCtrl to SHEStand, through other emphysematous groups) whereas anticoagulant factor, AT, shows the obverse tendency.

**Figure 3:**
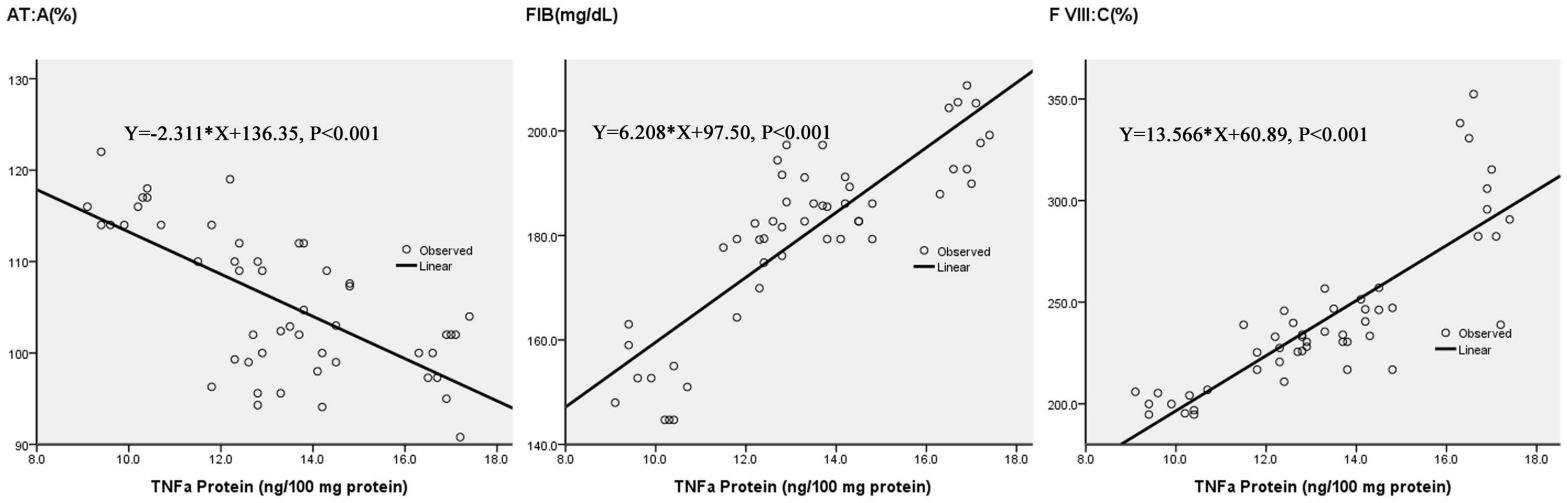
AT correlates with TNFα protein negatively. FIB and F VIII correlate with TNFα protein positively.

From the relatively high correlation coefficients seen in Table 3, inflammatory cytokines of liver correlate very well with coagulant factors positively and with AT negatively. Figure 3 illustrates these correlations clearly. With the increasing of TNFα protein, FIB and F VIII increase gradually and AT decrease obviously. Correlation coefficients for TNFα protein with FIB, F VIII and AT are 0.853, 0.837 and −0.684, respectively, all *P* values <0.01.

**Table 3:** Correlations between inflammatory parameters and coagulant factors/AT from liver.

	AT	FIB	F VIII	Score for liver	IL-6mRNA	IL-6protein	TNFαmRNA	TNFαprotein
**AT**		−0.713**	−0.562**	−0.742**	−0.720**	−0.534**	−0.738**	−0.684**
**FIB**			0.683**	0.921**	0.855**	0.689**	0.891**	0.853**
**F VIII**				0.709**	0.773**	0.866**	0.807**	0.837**
**Score for liver**					0.870**	0.695**	0.930**	0.867**
**IL-6 mRNA**						0.748**	0.968**	0.934**
**IL-6 protein**							0.768**	0.830**
**TNFα mRNA**								0.945**
**TNFα protein**								

Linear correlation analysis coefficients are shown.

** *P*<0.01.

## Discussion

Our model of emphysema, caused by smoke exposure and identified with pathologic criteria, reflects partly the pathobiologic features of emphysematous COPD which is induced commonly by inhalation of noxious gasses and particulate matter resulting in a chronic persistent inflammation in the lung [34]. This kind of chronic inflammatory response of the lung in some COPD patients may be associated with a significant systemic inflammatory response with downstream adverse clinical health effects, such as cardiovascular diseases, osteoporosis, diabetes, metabolic syndrome [1] and increased risk of hospitalization and mortality and is therefore a topic of increasing concern [35]. It has even been suggested that COPD might be a part of a chronic systemic inflammatory syndrome [36]. As the most important metabolic organ, most inflammatory materials are synthesized in liver. In a literature, it has been implicated that in COPD patients inflammatory mediators generated in the lung may “spill over” into the circulation and then activate the liver to release inflammatory proteins, which may be the real detrimental factors to target organs and the resource of systemic inflammation. Liver may play an important role in the COPD systemic inflammation chain [37]. Not only a pro-inflammatory status, COPD may also be in a pro-thrombotic status. There is an increased risk of thrombotic events occurs in those with COPD and our concerned coagulant factors, AT, FIB and F VIII, have all been influenced by COPD [38], [39]. In a cohort study of over 23,000 patients, Curkendall [40] reported an increased risk of acute myocardial infarction, stroke and pulmonary embolism in COPD. In a 5,451-patient deep venous thrombosis (DVT) registry, 668 (12.3%) had COPD as a comorbid condition [41]; and in 197 consecutive COPD patients, prevalence of pulmonary embolism was about 25% [42]. Though thrombosis is a multifactorial state and involves various processes such as platelet activation, endothelial cell injury, and activation of coagulation-fibrinolysis system, a hypercoagulative state may also be implicated with the increasing of these 3 hepatic factors we studied in the clotting system. Additionally, although it normally presents a nonthrombogenic surface, endothelium is capable of procoagulant activity and suppression of native anticoagulant properties. Exposure to hypoxic environments typically shifts the endothelial phenotype toward that in which anticoagulant characteristics are reduced and proinflammatory features dominate the endovascular milieu, therefore may increase procoagulant activity [43], [44] and inhibit fibrinolysis [45]. Inflammation that comes from hypoxia can promote the assembly of the prothrombinase complex and subsequently generates thrombin [46]. A study of healthy volunteers showed a transient increase in markers of coagulation activation during exposure to hypoxia [47]. Although these studies are done in vitro or in healthy volunteers, which may be unethical in COPD patients, further hypoxia, SH as an example, may worsen the preexisting pro-coagulant state in COPD as well putatively.

According to ABG results obtained from preliminary experiments and pathologic characteristics of smoke exposure rats, we have made a rat model of SHE through simulating the status of SH (with exposing rats to hypoxia during sleep period, as diagnostic criteria from ICSD-2 [4]–[7]) in emphysematous rats (caused by exposure to cigarette smoke for a long period of time and identified with pathologic criteria [31]–[33]). Our SHE model, in fact, is the “combination” of emphysema and SH, not a real COPD sleep related hypoxia (SRH) which appears at the end stage of COPD. In the real COPD SRH pattern, SRH is secondary to COPD, not combined by an exogenous hypoxia environment. As a rat model, for building a uniform hypoxia severity and time period, our exposure pattern may be a reasonable and feasible methodology and it does represent some of the pathological characteristics of SRH.

The coagulant factors (including vWF, a binder and protector of F VIII) and inflammatory parameters we measured are higher in emphysematous groups than those in SHCtrl when AT is higher in SHCtrl, indicating that, at least compared with simple SH, the emphysema (combined or not combined with SH) caused by smoke exposure may lead to a more severe inflammation and more obvious prothrombotic status. Among emphysematous groups, shown in table 2 and more clearly in Figure 2, inflammatory and prothrombotic tendency is significantly enhanced by SH, which is the evidence of synergism of emphysema and SH in this pathologic pathway. Because SHEStand has more severe inflammation and coagulabilty than SHEShort and SHEMild, we may conclude a dose-dependent mechanism in this synergism. In similar emphysema extent, the lower and longer time period of SH exposure, the more significant this synergism is.

In our study, inflammatory parameters of the liver correlate well with coagulant factors positively and with AT negatively. The potential influence of inflammation on coagulation may contribute further to thrombogenesis, while abnormal blood coagulation contributes directly to the pathophysiology and mortality of COPD, which means a more severe inflammatory state and a vicious cycle may be created [29]. Figure 3 illustrates and quantifies these correlations; it is clearly shown that with the increasing inflammation there may be an increase in blood coagulabilty.

As above mentioned, this SHE model represents some of the characteristics of SRH. In SRH, alveolar hypoventilation appears to play a major role, especially during rapid eye movement (REM) sleep. This was demonstrated by Becker [48] in a study of nine patients with underlying COPD. Compared with wakefulness, minute ventilation decreased 16% during non-REM sleep and 32% during REM sleep, predominantly because of a decrease in tidal volume, measured with a pneumotachograph. Additionally, arterial hypoxemia alone may be the product of worsening ventilation-perfusion mismatch with a greater effective shunt [5]. Alveolar hypoventilation occurs predominantly during REM sleep. Ventilation-perfusion mismatch can occur during non-REM sleep episodes as well [8].

Fletcher [49] showed that 27% of 135 patients with COPD with awake PaO_2_ exceeding 60 mm Hg had SRH. In another study, up to 25% of patients with COPD exhibit SRH, despite having a daytime PaO_2_ above 60 mm Hg [50]. Koo [51] found a mean decrease in PaO_2_ of 13.5 mm Hg and a mean increase of PaCO_2_ of 8.3 mm Hg during REM sleep in COPD subjects. In a study of 16 COPD patients [7], the mean SaO_2_ was 90±6% and the lowest SaO_2_ during the night was 83±8%. The percentage total sleep time (TST) with a SaO_2_ less than 90% was 37±45%. Sergi [12] found that SRH in COPD patients may represent an independent risk factor for the development of chronic respiratory failure in COPD patients with day-time PaO_2_ >60 mm Hg. Fletcher [13] noted significantly decreased survival among patients with SRH compared with those without. In a study focused on the systemic effects of nocturnal hypoxemia in COPD patients, the number of patients presenting C reactive protein (CRP) levels above those considered normal was significantly greater in the desaturation group which is the evidence of pronounced inflammatory activation in COPD patients with nocturnal hypoxemia. [16]

The necessity of treatment of isolated SRH in COPD has been debated for many years [52]. American Thoracic Society guidelines [53] for diagnosis and treatment of COPD recommended increasing oxygen flow by 1 L/min during sleep in patients undergoing long-term oxygen therapy (LTOT) to prevent nocturnal oxygen desaturation. However, those recommendations were not supported by some related studies. [9], [54], [55].

Through our study we can infer a potential pathologic pathway. In our emphysematous rat model, inflammatory mediators in liver increase and subsequently more coagulant factors are produced and AT is suppressed. When SH is combined with emphysema, synergistic effects appear, and the inflammation and coagulability may enhance each other and produce a more significant inflammatory and prothrombotic status which will lead to further health problems, as its clinical implication. In conclusion, the authors believe that along with optimal treatment for COPD, therapy for increasing PaO_2_ to >60 mm Hg is also important for the improvement of inflammation and coagulation, no matter what method is used (LTOT, nocturnal non-invasive positive pressure ventilation (NNIPPV), or medications).
